# "Smoking in Children's Environment Test": a qualitative study of experiences of a new instrument applied in preventive work in child health care

**DOI:** 10.1186/1471-2431-11-113

**Published:** 2011-12-15

**Authors:** Noomi Carlsson, Siw Alehagen, Boel Andersson Gäre, AnnaKarin Johansson

**Affiliations:** 1 Department of Clinical and Experimental Medicine, Division of Paediatrics, Faculty of Health Sciences, Linköping University, SE-581 83 Linköping, Sweden; 2 Department of Public Health and Medical Care, Jönköping County Council, Box 1024, SE-551 11 Jönköping, Sweden; 3 Department of Medicine and Health, Division of Nursing Science, Faculty of Health Sciences, Linköping University, SE-581 83 Linköping, Sweden; 4 Futurum - the Academy for Healthcare, Jönköping County Council, SE-551 85 Jönköping, Sweden; 5 The Jönköping Academy for Improvement of Health and Welfare, Jönköping University, Box 1026, SE-551 11 Jönköping, Sweden

## Abstract

**Background:**

Despite knowledge of the adverse health effects of passive smoking, children are still being exposed. Children's nurses play an important role in tobacco preventive work through dialogue with parents aimed at identifying how children can be protected from environmental tobacco smoke (ETS) exposure. The study describes the experiences of Child Health Care (CHC) nurses when using the validated instrument SiCET (Smoking in Children's Environment Test) in dialogue with parents.

**Method:**

In an intervention in CHC centres in south-eastern Sweden nurses were invited to use the SiCET. Eighteen nurses participated in focus group interviews. Transcripts were reviewed and their contents were coded into categories by three investigators using the method described for focus groups interviews.

**Results:**

The SiCET was used in dialogue with parents in tobacco preventive work and resulted in focused discussions on smoking and support for behavioural changes among parents. The instrument had both strengths and limitations. The nurses experienced that the SiCET facilitated dialogue with parents and gave a comprehensive view of the child's ETS exposure. This gave nurses the possibility of taking on a supportive role by offering parents long-term help in protecting their child from ETS exposure and in considering smoking cessation.

**Conclusion:**

Our findings indicate that the SiCET supports nurses in their dialogue with parents on children's ETS exposure at CHC. There is a need for more clinical use and evaluation of the SiCET to determine its usefulness in clinical practice under varying circumstances.

## Background

Despite knowledge of the negative impact of environmental tobacco smoke (ETS) exposure on non-smokers' health [1], children are still being exposed to ETS, most often by their parents [2]. The adverse impact of ETS exposure on the health of children is significant and increases the risk of middle ear infections, pneumonia, wheezing, coughs, bronchitis, bronchiolitis, impaired pulmonary function and sudden infant death syndrome [3,4].

Interventions have been carried out in different parts of the world in order to protect children from ETS exposure [5]. Sweden has a long tradition of preventive work in the tobacco field, in particular concerning smoke-free environments. According to Swedish tobacco law smoking is prohibited in environments frequented by children including restaurants, nurseries and schools. Legislation and increased knowledge of the risks associated with passive smoking have changed the social norm in Sweden regarding where smoking is considered acceptable in the community. Nowadays, the home is the only place where smoking is considered acceptable [6]. Interventions regarding the importance of protecting children from ETS exposure have been implemented in Child Health Care (CHC) in Sweden [7,8] but they have failed to reach socio-economically disadvantaged groups [9]. These groups are, according to CHC nurses, difficult to reach in tobacco preventive work [10] and they also have the highest prevalence of smokers.

In 2009 about 12 % of the adult population of Sweden were daily smokers [11] and about 13% of children born in 2007 had at least one family-member who smoked in the home during their first month of life. At 8 months of age, 14 % of children had at least one smoker in their immediate family [12]. According to an earlier study, approximate 60% of smoking parents claimed that they only smoked outdoors [6].

Smoking with water-pipes is a fairly new phenomenon in Sweden. According to a survey conducted by the European Union in 2009, 27% of the Swedish population (15 years of age and older) has tried smoking through a water-pipe [13]. Daher et al. showed that a one-hour water pipe smoking session produces as much passive smoke, with all its harmful substances, as four smoked cigarettes. Smoking through a water-pipe also produces carbon monoxide which is equivalent to 20 cigarettes worth of passive smoke during the same period [14]. Smoking through water-pipes has thus become an important issue in the context of both children and their parents.

CHC professionals in Sweden are expected to detect children's ETS exposure during appointments with parents. According to the Swedish National Council on health promotion activities related to ETS exposure, CHC nurses are expected to ask parents if any smoking takes place in the home [15]. The parents' response to this question is registered in the child's health record when the child is 0-4 weeks, 8 months, 18 months and 4 years of age. CHC nurses play an important role in tobacco preventive work. Previous Swedish studies, however, have shown that parents have not been satisfied with tobacco preventive work carried out at CHC centres [16,17]. One study showed that 56 % of parents surveyed stated that CHC nurses only registered if parents were smokers in the medical record, refraining from discussing the matter further [17].

One review article showed that there is not sufficient evidence to show which interventions are the most effective in decreasing parental smoking and minimizing children's ETS exposure [18]. Interventions designed to take into account an individual's current stage of change (or readiness to change a health-related behaviour) would be more effective and efficient than "one size fits all" interventions [19]. Motivational Interviewing (MI) has proven to be useful in interventions for smoking cessation. With this approach dialogue is adjusted according to the individual's current stage of change [20].

One instrument that has proven to be useful and has shown to have good effect on behavioural changes is the health curve. This instrument supports dialogue on the individual's life style and is used in both CHC and primary care [21,22]. The instrument AUDIT-C (Alcohol Use Disorders Identification Test) is used by midwives in antenatal care to support dialogue on alcohol consumption with pregnant women [23]. We wanted to develop a similar instrument to support dialogue on smoking in CHC centres with regard to the ETS exposure of children.

The SiCET (Smoking in Children's Environment Test) instrument used in this study is a modified version of the instrument developed by Johansson et al [24] regarding children's ETS exposure. It was designed to measure children's ETS exposure and also to support CHC nurses in their dialogue with parents on their smoking behaviour in children's environments. This instrument has been used to investigate the significance of parents' smoking behaviour on children's ETS exposure as it allows the described smoking behaviour of the parents to be related to the level of cotinine in the children's urine. The instrument is also designed to be used in consulting situations in clinical settings as the parents' responses could serve as valuable guiding factors during counselling. The instrument has not been previously tested for this purpose in any clinical setting.

The aim of this study was to describe how nurses working in CHC centres experienced using the SiCET instrument in tobacco preventive work in CHC.

## Method

An exploratory qualitative approach with focus group interviews (FGI) was chosen for this study. The use of focus groups is considered an effective method of assessing needs, testing new programmes and ideas and also improving existing programmes [25]. Using this method, data is produced through the interaction of the group and an understanding of the experiences, opinions and attitudes of the participants is generated [25,26]. The size of groups can vary depending on the topic but a range of 4-15 participants is commonly recommended [25,27]. Smaller groups are recommended for studies aiming to gain an understanding of experiences [25]. The number of groups is often suggested to be 3-5 [27]. The goal of this study was to determine the variability of experiences of an instrument [25].

### The Instrument - SiCET (Smoking in Children's Environment Test)

The instrument comprises ten issues and answers by choice of different responses: number of smokers in the household, cigarette consumption in the home on weekdays and weekends and which strategies for ETS protection have been used as well as their frequency of use. The importance of smoking in different places in the home is also included. Finally, the frequency of the child's exposure to ETS outdoors is included as is the stability of smoking habits in the home [24]. The instrument was modified for this study and was named SiCET. The alternative water-pipe was added to the question concerning smoking tobacco other than cigarettes. Two new questions were added to the instrument regarding if the child's grandparents smoke and if there is anything the parents would like to change in order to protect their child from ETS exposure. The alternative "in the car" was added to the question where parents were asked to specify where smoking is carried out. Parents also had the possibility of writing their own alternatives and comments. The instrument was validated for parents living in Swedish conditions. To facilitate the use of the instrument when meeting immigrant families, the instrument was translated into 9 languages; English, Spanish, Albanian, Bosnian, Serbian, Vietnamese, Cantonese, Somali and Arabic. These languages were identified as the languages spoken in the CHC areas included in the study.

The nurses were asked to use the instrument with families with both newborn and older children. The parents' responses to questions could then be used in further dialogue on the subject of smoking and ETS exposure.

### Setting and sample

Twenty-two CHC nurses who were taking part in an intervention for tobacco prevention in CHC in a county in the south-eastern Sweden accepted the invitation to participate in this study. Eighteen of these nurses participated in scheduled FGIs. The FGIs were conducted in five different CHC centres in the county. The nurses' working locations were taken into consideration when scheduling the interviews. Avoiding long travelling made participation possible. All participants had experience of using the SiCET primarily from working within the intervention project which had been running for a period of six months at the time of the interviews. All participants were trained in using the method Motivational Interviewing [20,28]. The majority of participants had long experience of working in CHC (Table 1) and all participants had permission from their supervisors to participate in the study.

**Table 1:** Characteristics of the study population

Variables	Paediatric Nurse	District Nurse	All Nurses
Education *	5	13	18
Age (years)**	47.5 (32-60)	47 (36-58)	47 (32-60)
Experience of work in Child Health Care**	9 (1-35)	8 (1.5-33)	9 (1-35)

* Number of nurses

** Values are median (range)

### Focus groups interviews

We chose to use a questioning route which encourages free expression, according to Kreuger [25], using open-ended questions in the FGIs. The questions were developed by the research team and discussed with other researchers experienced in the method and one minor revision was made. The questioning route began with some introductory questions concerning names, location of workplace and experience in tobacco preventive work. The open-ended key questions which followed focused on 1) the SiCET form with its various issues, 2) the parents' response to the questionnaire, 3) the SiCET as a support in dialogue and 4) the use of interpreters. Finally the nurses were asked if there were any changes they wanted made to the questionnaire and about their opinions on SiCET as an instrument in tobacco preventive work in CHC.

A pilot FGI aiming to test the interview guide was performed by the first author (NC) and an assistant moderator. Five nurses participated in the pilot FGI. The content of the pilot FGI was of good quality and was therefore included in the analysis.

The participants were divided into five groups according to the location of their workplaces in the county. The FGIs were led by the first author (project-leader) who took the role of moderator which involved guiding the group through a discussion using open ended questions [25]. A public health planner experienced in working with focus groups served as an assistant moderator, made field notes and took care of practical issues. The FGIs were carried out in different CHC centres within a two week period during September 2010. Each FGI included between two and six participants and consisted of 32 to 45 minutes of active discussion following the questioning route. Participants interacted with each other and as the FGIs continued this interaction developed in a positive direction. The FGIs were audio-taped and transcribed verbatim by the first author (NC).

### Data analysis

The FGIs were analysed in accordance to the method described for focus groups by Kreuger and Casey [25]. Immediately after the interviews, they were transcribed by the first author. This started the analysis process. The transcription was then read several times to obtain a comprehensive picture and deeper understanding of the interviews. All opinions of the use of the SiCET were marked with a highlighter. Memos were written in the margins. Two of the authors (NC, SA) sorted the content of the interviews into categories which were thoroughly examined and discussed. Subcategories were created for composite sections of the text with similar meanings and discussed by the authors. A third author (AKJ) read the interviews independently and identified categories and subcategories separately. The results were discussed by the three authors (NC, SA, AKJ) and when no new interpretations were found the names of the categories and subcategories were decided and kept as close to the content of the original text as possible. The remaining material which was considered irrelevant to the study was then excluded. The triangulation resulting from involving the third author (AKJ) strengthened the dependability of this process [25].

### Ethical approval

No local research ethics committee in Sweden has received this study for assessment because this is not necessary under Swedish law when patients are not involved or affected. The study was designed and implemented to maintain autonomy and integrity according to the common principles in human subject research [29]. Participants were informed orally and in writing that their participation was voluntary, that they could discontinue participation at any time and that all data collected would be treated confidentially.

## Results

The analysis emerged into three categories and seven sub-categories (Table 2). Verbatim quotations are used to illustrate the findings and show a link to the original data.

**Table 2:** Summary of the categories and sub-categories extracted from the focus group interviews.

Categories	Sub-categories
SiCET in dialogue	Direct dialogue
	Indirect dialogue
	Dialogue and interpreters
	
The SiCET instrument	Strengths
	Limitations
	
SiCET and its outcomes	Focused discussion on smoking
	Support for changes

### SiCET in dialogue

The nurses perceived that the SiCET facilitated dialogue with parents regarding tobacco smoke exposure and three sub-categories were generated.

### Direct dialogue

In direct, face to face dialogue with the parents, the nurses discussed the subject of smoking on more than one occasion. It was possible to broach the subject at a number of the scheduled visits in CHC centres during the child's first year of life. The nurses felt that it was natural to talk about the child's ETS exposure using the parents' answers in the SiCET as a basis for the discussion. Parents were free to talk about what they had written in the form. The nurses could ask further supplementary questions which led to more in-depth discussions as several perspectives were highlighted.

One nurse said:

"The most important aspect (of using the SiCET) is that it helps initiate the dialogue I think...it's not just like...how is it, does anybody smoke in the home...and then you don't know what to say... it really assists in having an open discussion."

Another stated:

"There are more discussions (when using the SiCET)...you can see things from different perspectives."

Conflicts could sometimes arise between parents if they had different views about which environments smoking takes place in. The nurses experienced themselves as moderators in these conflicts, by leading the discussion so that an accurate picture could emerge and the parents could reach consensus regarding the child's ETS exposure.

One nurse said:

"......the father, who was a smoker, said that he did not smoke in the car, but then the mother protested and said that he did ....that he should admit it ... and he said finally that he had smoked in the car occasionally, but this was not information which he would have given by himself."

### Indirect dialogue

In some cases conversations based on the SiCET involving only one parent resulted in both the parents discussing the subject with each other at home. In this way the nurses experienced that they were working indirectly with the absent parent. In cases where the father, who was the smoker, did not come to the CHC centre, mothers said that they had later taken up the subject of ETS in the home with the father. Then the mother could inform the father of appropriate places to smoke or try to motivate the father to quit. If the mother was motivated in trying to persuade the father to be smoke-free she could then function as an influence on the father.

One nurse said:

"I mean, if a non-smoking mother is motivated to help the father to quit smoking it can be quite influential at home... .. because they tell them .... now, you have to go outdoors and smoke ... .. motivating them to stop or cut down ... we' re not there every day to push them and remind them or support them or tell them what to do."

Even though the nurses were aware that this indirect dialogue took place, they were still dissatisfied due to not being able to have direct dialogue with the smoker in the family. In some cases the nurses offered to telephone the father but they were always met with excuses and these conversations never materialised. Some nurses, however, spoke about mothers who were not concerned about the fathers smoking and in these cases it was found to be impossible to influence the fathers at all.

One nurse said:

"I have met several women who explain away the problem...they say he smokes so little it doesn't matter."

In cases where it was found that relatives, often grandparents, were smokers, the nurses experienced that the parents wanted to discuss the matter with the aim of protecting their child. Children who were picked up by grandparents from pre-school could spend quite a lot of time in their grandparents' home environment. If the grandparents were smokers the nurse was able to provide support to the parents to help them in their ambition to create a smoke-free environment outside the child's home even if it was a delicate issue to deal with.

One nurse said:

"I'm not sure if I would have found out about it (without the SiCET)... I might have known that the child was with that the grandmother but I might not have known if the grandmother was a smoker or not. I probably would not have known that....I'm not sure if I would have asked or not...if it would have occurred to me or not."

There were also situations where an indirect dialogue would have been desirable but was not realized. Even though the nurses had some knowledge of and were eager to talk about the smoking habits of the parents' relatives and friends many parents felt it too private and therefore had problems discussing the issue.

One nurse said:

"I visited one family where the father smoked outdoors and the mother said that it was difficult when relatives and friends visited as they were used to smoking indoors. The mother could not bring herself to tell them that they had to go outdoors to smoke. She said she could never, ever say those words."

### Dialogue and interpreters

The nurses used interpreters to varying degrees in dialogues with parents. Several of them felt it was easier to work without an interpreter if one of the parents could speak Swedish. The nurses' use of interpreters when using the SiCET also varied because it was the parents who decided if an interpreter was necessary when visiting the CHC centre. Some nurses used physically present interpreters, while others used telephone interpreters.

One nurse commented:

"Some of the Somali women don't want to have an interpreter in the room."

According to some nurses, using a physically present interpreter had both advantages and disadvantages. The advantages included that the interpreter could see the SiCET with its layout and questions and could therefore better understand what the dialogue was based on. The disadvantages included that there sometimes exists a relationship between the interpreter and the parents which can complicate the dialogue. Furthermore, the interpreter's body language and clothing could indicate a social discrepancy between him/herself and the parents. Another nurse expressed concerns that a parent might not trust the interpreter and would therefore not be honest in the dialogue.

Another nurse commented:

*"...if the interpreter is a little bit proper and formal. ...and the family, we can say they are more basic in the way they talk ... it can be difficult with an interpreter who sits there nicely like a princess and maybe says just the right words but she is a person who does not belong there ... that does not fit into the context ... I have experienced this sometimes when using an interpreter*."

When the nurses were working with a physically present interpreter they used the SiCET translated into the parents' native language. This made it easier both for the non-Swedish speaking parent and the interpreter. Most of the nurses experienced working with interpreters as extremely time-consuming. On one occasion a parent had become irritated because of all the questions placed through the interpreter. On some occasions telephone interpreters were used. One nurse only used telephone interpreters and thought it worked well despite the interpreter not having the possibility of following the questions in the SiCET. The nurse read through the SiCET sentence by sentence and the interpreter translated for the parents.

However, one nurse said:

"There are no optimal conversations using telephone interpreters ...I think it is difficult."

Another commented:

"It depends on who is interpreting...some interpreters are excellent."

A third nurse said:

*"We use almost only telephone interpreters here and I think it works well."*

The instrument SiCET

The nurses' experiences of the SiCET as an instrument generated in two sub-categories. The advantages outweighed the disadvantages and the nurses were positive towards the continued use of the SiCET in their tobacco preventive work after the end of the study.

### Strengths

One strength of the SiCET that was stressed by the nurses was its limited number of questions. This enabled parents to respond to the questionnaire relatively quickly. No parent had been hesitant to answer any question. Another strength was that the SiCET was a validated instrument which gave the nurses a feeling of confidence when it was used and they felt legitimacy in using the form. The nurses saw the SiCET as a professionally developed, objective instrument.

The nurses experienced that the detailed questions helped them to broaden the concept of environment and dialogues with parents were more open and honest than in unstructured discussions. They compared the SiCET with other validated forms and stressed how it simplified working with difficult issues. Another strength was that parents with non-Swedish backgrounds were able to use the SiCET in their own language. The SiCET made it easier for the nurses to work in a more structured and sustainable way.

One nurse said:

"It is more legitimate...to ask these questions when you have this paper."

Another nurse claimed that her role became more neutral when using the SiCET:

"It is not me who is curious; it is the paper I am holding."

The usefulness of the instrument was experienced as high, and some nurses suggested that it should be used in other professional areas of health and medical care such as in antenatal care and in paediatric clinics. This would then provide parents with the same information throughout the entire health-care chain. The nurses pointed out the possibility of it being used continuously to follow the child's ETS exposure. In addition, the nurses considered the SiCET with its in-depth questions as a signal to parents that ETS is an important issue which concerns the child's whole environment.

One nurse said:

"I think that the SiCET could be used even before the birth of the child in antenatal care ..... conduct a survey already there ... follow this issue through the whole health-care chain."

### Limitations

One limitation was that non-Swedish parents sometimes needed support in understanding the questions, even if they were using a version translated into their own language. When the parents used the translated version the nurse had to fill in a Swedish version at the same time, to avoid difficulty in understanding the parents' answers. For example that Arabic is read from right to left created some difficulties.

One nurse commented:

"They maybe feel a bit silly asking about the questions...and so then they fill in the form in their own way."

The question asking about how often the child is in environments outside the home where smoking occurs had sometimes led to discussion. Parents were often unsure about what is considered an environment outside the home, if this includes both indoor and outdoor environments.

One nurse said:

"They have asked about the question concerning how often the child is in an environment outside the home where smoking occurs ...many parents think this is difficult to understand ... almost everyone has asked if it includes the outdoors."

Some of the nurses had to explain the difference between two fairly similar questions to some parents. This confusion was over the questions asking where smoking occurred in the home and how important it was for the individual to smoke in these places.

### SiCET and its outcomes

The outcomes of using the SiCET generated two sub-categories, focused discussion on smoking and support for changes.

#### Focused discussion on smoking

By using the SiCET, nurses could provide support for both smoking parents and non-smoking parents in discussions on how to create a tobacco-free environment for the child.

Overall, the nurses felt that the SiCET supported them in dialogues with the parents and it helped to bring up many specific issues about tobacco which might not have been discussed otherwise. The children's environment, both indoors and outdoors, became a natural topic in the conversation. Other issues discussed were smoking in bus shelters, cars, basements, garages, friends who smoked and clothes smelling of smoke. Questions about smoking with a water-pipe added a new dimension to the discussion. The SiCET thus provided the nurses with a complete picture of the child's environment on which to base further discussions.

Nurses experienced that discussions concerning tobacco with parents were more satisfactory when using the SiCET as it made in-depth conversations about smoking in the child's environment more obtainable. The smoking status and smoking frequency of the parents could be discussed in an easier manner.

One nurse said:

"It has become easier during home visits also...I don't ask if they smoke ... instead I ask if there is anyone close to the child who smokes...the questions are wider and more encompassing, I think."

Another said:

"If I think it's a bit difficult and I sense that they are reluctant to talk about it, then I think it is helpful...that I have the paper with me (the SiCET)."

Support for changes

The nurses described the use of the SiCET as a process in different phases with which they could support the parents in their changing process. When the parents realised the significance of their child's ETS exposure it led to reflections and thoughts about how to make changes to protect the child.

One nurse said:

"It starts a thinking process in the person completing the form. ... .. it leads to something happening."

While another nurse said:

"But it doesn't feel like they fill in the question on making changes because they **have to **...no it doesn't feel like that...it feels like they really want to make changes if they can....and that they want to learn how."

Some of the nurses used the method Motivational Interviewing (MI) to make progress with parents and support them in smoking cessation or making changes in their smoking behaviour. Some fathers who smoke thought of specific improvements they could make instead of quitting smoking, such as changing clothes after smoking on the balcony.

One nurse commented:

"I think the question asking if they want to make changes to protect their child is the most important or the most interesting for me, actually. If they answer "absolutely" then I have a starting point and if they answer "no" then I know where they stand ...this is the question I look at before I start discussing the other questions."

There were also ambitions among the parents to support each other during proposed lifestyle changes.

One nurse said:

"...one mother has even offered ... she does not smoke...but she offered to stop eating candy... and that they support each other in that way...maybe there's a will to make changes there."

If a parent wanted to quit smoking, the change question on the SiCET was a reminder for the nurse to write a referral to a professional for smoking cessation expert. If they didn't want to quit smoking the nurses were told about the parents' behavioural changes such as moving their smoking from indoors to outdoors. Some smokers had also reduced the number of cigarettes smoked each day. Parents were proud of the changes they made even if they didn't quit smoking. It was like an acknowledgement that it had given results.

As one nurse said:

"She looked at her smoking status which wasn't good and it made her think about it and then quit...or almost quit anyway. There is a big difference between smoking one cigarette once a week compared to, well I can't really remember if it was 10 cigarettes daily indoors or something like that."

Nurses also experienced that changes from one day to another were not often possible so they had to accept long-term thinking on the part of the parents. The nurses thought that even if there were no changes made after the first occasion when the SiCET was used, that changes could occur in the future if they used the SiCET again in meetings with the family.

One nurse said:

"Yes, at eight months you need to write about it in the health record so it would work well if we use the SiCET when they are newborns and the parents are smokers and then we could use it again when the child is eight months of age."

## Discussion

The CHC nurses experienced that the SiCET provided some support in opening conversations with both smoking and non-smoking parents so that a natural dialogue could take place concerning tobacco preventive work. The SiCET provided both the parents and the nurses with a picture of the child's ETS exposure. This led to increased understanding of the child's actual situation which could facilitate behavioural changes among parents where the nurses could play a supporting role. According to the nurses conversations were more in-depth and constructive when using the SiCET.

A previous study found that nurses experienced difficulties in getting responses from parents in dialogues on smoking [10]. Our findings show that the SiCET facilitated dialogues and led to more detailed and in-depth conversations. This shows the strength of the SiCET as an instrument as it helps open the conversation and parents themselves can continue to talk using their answers to the questions as a base. Another instrument, Alcohol Use Disorders Identification Test (AUDIT), which is used in antenatal care for improved detection of women with high-risk alcohol consumption, is not shown to have the same positive effect in dialogue between the midwife and the mother [30]. This is despite the fact that it was intended for use as both a screening instrument and a communication tool which could be used to provide a structure on which to base conversations about alcohol [31].

Some nurses in this study experienced parents disagreeing with each other on how they should respond to questions about their smoking behaviour in the child's environment. The nurses experienced themselves as moderators in these conflicts and they assisted the parents in reaching a common view. Many studies using objective markers such as cotinine have shown that smokers are usually honest in their responses on smoking behaviour [6,32,33] with the exception of one study [34].

According to the nurses, the most rewarding conversations occurred when both parents were present. Contacting the father in cases where he did not accompany the mother to the CHC centre was experienced as problematic. Nurses offered to contact the fathers via telephone in an attempt to help, but this never materialised. Nurses found this to be dissatisfactory and ways to avoid indirect dialogue need to be developed. Nurses could have been less conservative in their actions and insisted on talking with the fathers directly as it has been shown that a more active approach is required to reach smoking fathers. Telephone contact is one possible method of having a direct dialogue with fathers and this has been supported by a study where proactive telephone counseling was found to be a successful approach for work with tobacco cessation among parents of young children [35]. More intensive efforts need to be made since both parents are entitled to the same information through direct contact with the nurse. A study by Fagerskiold [36] indicated the importance of father involvement in the child's upbringing as many fathers of today want to take an active role in child care and have more communication with the CHC nurses.

Asking people about their smoking behaviour is a delicate issue and can be perceived as intrusive. The professional asking the question might think it is too private, but a study shows that parents are often more willing to approach the issue of smoking than the professional expects [17]. The attitudes of the professionals might be a bigger obstacle than the clients' need for integrity. The SiCET showed a great strength in addressing this problem as it provides an overall picture of the environments in which children could be exposed to passive smoking. Approaching relatives and friends about their smoking behaviour was experienced as too difficult for some of the parents according to the nurses. This finding strengthens the parents' need for support from the nurses in creating a smoke-free policy in the home. In this process parents can then refer to facts given by the CHC nurses.

It is important to use an interpreter when meeting parents with another native language, as language difficulties could become a barrier in the dialogue [37]. Interpreters in health care have proven to be underused [38,39]. Interpretation takes time irritations may arise and preparation is required. All nurses in CHC have the possibility of using interpreters during their conversations with parents. One problem is to organise an interpreter to come to the clinic at short notice and another problem is the difficulty in getting an interpreter if the parents speak an uncommon language. Telephone interpreters can be organised at shorter notice, offer a wider range of languages [40] and are preferred by professionals [41]. However, the nurses who took part in this study did not all prefer telephone interpreters when using the SiCET. Dialogues using a telephone interpreter have to be prepared in advance to assure their success [42] . When using instruments such as the SiCET it is of great importance that the interpreter has access to both the Swedish version and the translated version of the instrument to avoid misunderstandings in the translation.

Due to the instrument providing the nurses with positive experiences in dialogue with parents the nurses emphasised the possibility of using the SiCET throughout the health-care chain. In antenatal care midwives could support parents and assist them in quitting smoking and in this way create a smoke-free environment for the expected child. In children with obstructive diseases where parents are smokers, the professionals in paediatric clinics could use the SiCET instrument to assist dialogue about the child's ETS exposure. In collaboration between professionals, it is possible to develop a strategy for situations in the health-care chain which would increase parents' confidence in the advice they receive in health care [43,44]. Professionals in the health-care chain could use the SiCET to motivate smoking parents to change their smoking habits or help them by writing a referral to a smoking cessation expert.

The interviews helped identify some weaknesses in the SiCET. Parents, irrespective of native language used, wondered what the difference was between the question asking where they smoked in the home and that asking how important it was for the individual to smoke in these places. This shows the importance of questions being clearly written. The nurses also have to be familiar with the form to avoid using it mechanically. Parents may not be familiar with filling out forms, regardless of their native language, and when uncertainties arise the nurses must have the knowledge required to be able to explain the questions clearly. Perhaps the nurses should have had clearer instructions before using the SiCET.

The SiCET, in its current and tested form, is available in ten languages. It is important that all dialogue participants can read the questions in their own language. The nurse is responsible for leading the discussion, but both parties need to understand the form. It would facilitate matters if Swedish was integrated into the translated texts to allow the nurse to help parents read it in their own language.

A Swedish study [6] indicated that smoking exclusively outdoors with the door closed is the best way for smoking parents to protect their children from ETS exposure. A challenge for the CHC nurses is to give advice on smoking outdoors during the winter months in Sweden. A certain degree of self-sacrifice is required and smoking outdoors can be uncomfortable during cold weather. Despite this, smoking parents often use the balcony for smoking and direct guests to do so too.

Smoking on balconies can be disruptive to non-smoking neighbours because of the smell of smoke and the inconvenience this causes. The ETS exposure caused by this is regarded as insignificant and a legal trial held in Sweden indicated that it is not generally possible to ban smoking on balconies by law [45,46]. However, the Surgeon General Report [47] in USA states that non-harmful levels of ETS exposure have not been found in any study.

Smoking with water-pipes is a relatively new phenomenon in Sweden and knowledge on this is still limited. For this reason this topic was included in the intervention. Immigration from Africa, Asia including China and India and the Middle East where smoking with water-pipes is traditional is the reason behind its introduction to Sweden [48].

The positive dialogue between the parents and the nurses made talking about changes possible and could also facilitate behaviour change among the parents. Motivational Interviewing (MI) is an effective method for initiating life-style changes [20,28]. Our results show that MI is well suited to use together with the SiCET. The nurses' open questions invited the parents to tell them about their situations and this is one of the aims of MI. Confirmations, reflections and summaries are important components in the MI dialogue and these are also important when using the SiCET . These components show empathy and encourage people to continue to explore their thoughts. According to the nurses dialogues based on the SiCET in combination with MI have led to behaviour changes among some of the parents that nurses in this study have worked with.

### Strength and limitations in the study

A strength in the study is that the SiCET has been tested over a six-month period in different CHC contexts distributed throughout the studied county.

A possible limitation was the focus-group sizes. In smaller groups, group dynamics and interaction are limited but the group members have more space to express themselves [49]. In this study the nurses had difficulties in allocating time for the interviews because they were to be carried out during working hours. To simplify the situation we made a geographic division of the nurses in the focus-groups instead of mixing the nurses from different parts of the county. Despite this, illnesses among the participants at the time of the FGIs led to small groups. We decided to use FGIs as participants generally feel more confident in group interviews compared with individual interviews and they get 'carried away' more easily in the group discussions.

The first author was both project manager for the intervention and moderator of the focus-groups which may have influenced the results. It might have limited the participants will to talk about negative aspects of using the SiCET, but it might also have been a strength as the moderator was well oriented in the study and could help to deepen the discussion with relevant questions.

## Conclusion

Our findings indicate that the SiCET supports nurses in their dialogue with parents on children's ETS exposure at CHC. There is a need for more clinical use and evaluation of the SiCET to determine its usefulness in clinical practice under varying circumstances.

## Competing interests

The authors declare that they have no competing interests.

## Authors' contributions

NC, SA, BAG, and AKJ designed the study. NC moderated the focus groups. NC, SA and AKJ analysed the findings. NC, SA, BAG and AKJ participated in the drafting of the manuscript. All authors have read and approved the final version of this manuscript.

## Pre-publication history

The pre-publication history for this paper can be accessed here:

http://www.biomedcentral.com/1471-2431/11/113/prepub
